# TGFβ signaling networks in ovarian cancer progression and plasticity

**DOI:** 10.1007/s10585-021-10077-z

**Published:** 2021-02-15

**Authors:** Asha Kumari, Zainab Shonibare, Mehri Monavarian, Rebecca C. Arend, Nam Y. Lee, Gareth J. Inman, Karthikeyan Mythreye

**Affiliations:** 1 Division of Molecular and Cellular Pathology, Department of Pathology, University of Alabama at Birmingham, WTI 320B, 1824 Sixth Avenue South, Birmingham, AL 35294 USA; 2 Department of Obstetrics and Gynecology-Gynecologic Oncology, University of Alabama at Birmingham, Birmingham, AL 35233 USA; 3 Division of Pharmacology, Chemistry and Biochemistry, College of Medicine, University of Arizona, Tucson, AZ 85721 USA; 4 Cancer Research UK Beatson Institute and Institute of Cancer Sciences, University of Glasgow, Glasgow, UK

**Keywords:** EMT, TGFβ, Ovarian cancer, Tumor microenvironment, Metastasis

## Abstract

Epithelial ovarian cancer (EOC) is a leading cause of cancer-related death in women. Late-stage diagnosis with significant tumor burden, accompanied by recurrence and chemotherapy resistance, contributes to this poor prognosis. These morbidities are known to be tied to events associated with epithelial-mesenchymal transition (EMT) in cancer. During EMT, localized tumor cells alter their polarity, cell–cell junctions, cell–matrix interactions, acquire motility and invasiveness and an exaggerated potential for metastatic spread. Key triggers for EMT include the Transforming Growth Factor-β (TGFβ) family of growth factors which are actively produced by a wide array of cell types within a specific tumor and metastatic environment. Although TGFβ can act as either a tumor suppressor or promoter in cancer, TGFβ exhibits its pro-tumorigenic functions at least in part via EMT. TGFβ regulates EMT both at the transcriptional and post-transcriptional levels as outlined here. Despite recent advances in TGFβ based therapeutics, limited progress has been seen for ovarian cancers that are in much need of new therapeutic strategies. Here, we summarize and discuss several recent insights into the underlying signaling mechanisms of the TGFβ isoforms in EMT in the unique metastatic environment of EOCs and the current therapeutic interventions that may be relevant.

## Introduction

Epithelial-mesenchymal transition (EMT) is a mechanism of trans-differentiation of epithelial cells into mesenchymal cells, wherein polarized epithelial cells alter their contacts with their neighboring cells, basement membrane and the surrounding tissues [1]. EMT is critical for embryogenesis and organ development as first reported in 1995 and since has been studied in great detail in both physiological and pathological conditions including wound healing, chronic disease with fibrotic changes and cancer progression [2, 3]. The presence of mesenchymal cells is associated with metastatic dissemination in cancer [4] as these cells can alter the extracellular matrix (ECM), and are more invasive and motile. On the opposite end of EMT is mesenchymal to epithelial transition (MET), which also occurs during development, wound healing, fibrosis and in cancer, where it may support metastatic outgrowth at distant sites [5]. Between EMT and MET, incomplete or partial EMT may occur, leading to hybrid states with simultaneous expression of epithelial (E) and mesenchymal (M) markers (E/M), observed in multiple cancer types [6, 7]. These intermediate E/M forms can differentiate into either mesenchymal cell types via EMT or revert back to an epithelial state by MET mechanisms providing a spectrum of plasticity opportunities to the cells [8]. The molecular and transcriptional networks regulating EMT are central to the process and are tightly regulated during both development and disease progression [2, 3]. Key transcription factors (TFs) that repress the epithelial phenotype act either directly (*SNAI1, SNAI2, SNAI3/SMUC, ZEB1 and ZEB2*) or indirectly (*TWIST1, TWIST2 and E2.2, GSC, SIX1, and FOXC2*) on E-box consensus sequences of E-cadherin’s promoter to repress E-cadherin expression [9–11] which has demonstrated itself to be a key event in EMT. As such, transcriptional control of EMT has been a subject of intense focus revealing multiple redundant, overlapping and tissue specific distinct roles for the TFs’ in this process. Most evident are studies on *TWIST* and *ZEB1* that have different roles in breast versus pancreatic cancers [12–16]. It is also increasingly apparent that EMT regulation is facilitated at multiple non-transcriptional levels including epigenetic, post-translational modifications of the TFs and their associated proteins, and via non-coding RNAs. In recent years, in-depth examination of the EMT-TFs and their analysis in vivo and in cancer patients has helped resolve some of the earlier controversies around EMT’s role in metastatic dissemination even in cancer cells or clusters of cells that lack so called EMT hallmark morphological differences [17, 18]. EMT-TF expression, and their activities themselves are regulated by extracellular stimuli, including but not limited to growth factors such as TGFβ and stressors such as inflammation and hypoxia [19, 20]. Such stimuli are tightly coupled to the specific tumor microenvironment (TME). Given the array of cellular and non-cellular components in each TME, unifying principles are challenging to develop and can only emerge upon a complete understanding of all TME components and their roles in a cancer specific manner. Much has been written about EMT and its relationship to TGFβ in cancer metastasis in the past decade [20–22], however in light of significant emerging knowledge on the cellular and acellular factors in the unique cancer microenvironments, new analysis is warranted. Towards this end herein, we focus on the role of EMT and TGFβ in the unique metastatic environment of ovarian cancers. Ovarian cancer follows a metastatic trajectory quite distinct from most other cancers. Patients continue to suffer from a lack of effective targeted therapies, despite the surge in EMT and TGFβ based therapeutic approaches for multiple tumor types.

## TGFβ family in ovarian and related cancers

### TGFβ superfamily

The discovery of the TGFβs’ can be traced as far back as 1976 when De Larco and Todaro first published in *Nature,* the presence of a group of proteins isolated from conditioned media of malignant tumors, that can induce morphological transformation in fibroblasts and promote growth of cells in soft agar. These proteins were called Transforming Growth Factor (TGF) now widely known as the TGFβ family [23]. Later, three isoforms of TGFβ (TGFβ1, TGFβ2, and TGFβ3) were characterized from this mixture [24–29] and were found to be encoded from individual genes located on different chromosomes [30, 31].The TGFβ superfamily is a large and expanding group of regulatory polypeptides. TGFβ1 is the archetype [32] with over 50 new members including TGFβ1-3, bone morphogenetic proteins (BMPs), growth and differentiation factors (GDFs), activins/inhibins (INHBA-E,INHA), and glial-derived neurotrophic factors (GDNFs), as well as Müllerian inhibiting substance (MIS), also referred to as anti-Müllerian hormone (AMH), nodal growth differentiation factor (Nodal), and left–right determination factor (Lefty) [33, 34] (Table 1).The TGFβ family members exist as either homo- or hetero-dimeric polypeptides, sharing a conserved cysteine knot structure [35]. All TGFβ isoforms are produced as a latent complex with an N-terminal latency-associated peptide (LAP) and secreted efficiently through interactions with latent TGFβ binding protein (LTBP) [36]. These complexes are rapidly stored in the ECM. Activation can then occur either by proteolytic removal of LAP or via cell-ECM generated forces by integrins (αvβ6 or αvβ8) interacting directly with conserved RGD motifs on the LAP of TGFβ1 and TGFβ3 [37–41] or indirectly via thrombospondin1 [42] and glycoprotein-A repetitions predominant (GARP) in platelets and regulatory T cells (Treg) [43]. Mature TGFβ dimers can then bind receptors to elicit signaling [44]. However not all TGFβs are created the same in terms of physiological functions and in pathologies. TGFβ1 knockout mice succumb to massive weight loss and death at three to five weeks of age due to uncontrolled systemic inflammation, causing lethal immune system defects and multiple inflammatory lesions [45]. Changes in endothelial cell differentiation and hematopoiesis was also reported in the yolk sac of TGFβ1 null mice [46]. The majority of TGFβ2 knockout mice also die before or shortly after birth due to developmental craniofacial defects and defects in the heart, lung, ear, eye, genitourinary tract, and skeleton. TGFβ3 null mice exhibit cleft palate and pulmonary developmental delays that result in early death [47, 48]. Although cleft palate and lung developmental delays were reported in both TGFβ2 and TGFβ3 null mice, the underlying mechanisms were shown to be different [49] with TGFβ3 specifically inducing EMT in the palate [50]. Evidence of cooperativity between the ligands also exists, as binding of TGFβ2 to its receptors is facilitated by TGFβ3 during certain physiological processes [51, 52] and in some contexts, TGFβ1 can cooperate with TGFβ2 to induce EMT [53].

**Table 1:** TGFβ receptors and SMADs’

LIGAND	Type II receptor	Type I receptor	Type III receptor	SMAD
TGFβ1	TGFβRII	ACVRL1/ALK1, ALK5, ACTRIA(ALK2)	TGFβRIII/betaglycan, Cripto	1,2,3,5,8
TGFβ2	TGFβRII	ACTRIA(ALK2), ALK5/ TGFβRI	TGFβRIII/betaglycan	1,2,3,5,8
TGFβ3	TGFβRII	ACVRL1/ALK1, ACTR-IA(ALK2), ALK5/ TGFβRI	TGFβRIII/betaglycan	1,2,3,5,8
BMP2/4	BMPRII, ACTRII, ACTRIIB	ACTR-IA(ALK2), BMPRIA (ALK3), BMPRIB (ALK6)	TGFβRIII/betaglycan, END/Endoglin, RGMB/Dragon	1,5,8
BMP5/6/7	ACTRII, ACTRIIB, BMPRII	ACVR1 (ALK2), BMPRIA (ALK3), BMPRIB (ALK6)	TGFβRIII/betaglycan	1,5,8
BMP9/GDF2	BMPRII	ACVRL1(ALK1), ACTR-IA(ALK2), BMPR-IA(ALK3)	END/Endoglin	1,5,8
BMP10	BMPRII	ACVRL1(ALK1)		
Activin A/B	ACTRII, ACTRIIB	ACTRIA(ALK2), ACTR-IB(ALK4)	END/Endoglin	2,3
Inhibin A/B	ACTRII, ACTRIIB	ACTRIB(ALK4), ACVRL1	TGFβRIII/betaglycan, END/Endoglin	
GDF5	BMPRII, ACT\\RII, ACTRIIB	BMPR1B (ALK6)	TGFβRIII/betaglycan	1,5,8
GDF1	ACTRII	ACTRIB(ALK4)	Cripto	2,3
GDF11	ACTRII, ACTRIIB	ACTRIB(ALK4)	N. D	2,3
MIS	MISRII	BMPRIA(ALK3), ACTRIA(ALK2), BMPRIB (ALK6)	N. D	1,5,8
Nodal	ACTRII, ACTRIIB	ACTRIB(ALK4), ALK7	Cripto	2,3
GDF9	ACTRII, BMPRII	BMPRIB (ALK6)	N. D	
GDF8	ACTRII, ACTRIIB	ActRIB (ALK4), ALK5	N. D	2,3
GDF6	BMPRII	BMPRIA (ALK3), BMPRIB (ALK6)	RGMB/Dragon	1/5/8

Immunohistological and in situ hybridization studies show overlapping spatiotemporal expression of all TGFβ isoforms in mouse embryos in cartilage, bone, muscle, heart, kidney, ear, eye, blood vessels, lungs, gastrointestinal tract, central nervous system, liver, and skin. No TGFβ3 protein expression was found in adrenal glands [54] indicating tissue specific distribution of the different isoforms of TGFβ. In the blood vessels, TGFβ1/2 were observed only in smooth muscles while higher levels of TGFβ3 are present in both smooth muscles and the endothelium [54, 55]. In the ovary, TGFβ2 and 3 are present during embryonic development [56] with TGFβ3 mRNA highly expressed in the ovarian surface epithelium (OSE) indicative of its possible pivotal role in the pathophysiology of the OSE [57]. Additionally, ovaries express TGFβ2 in the surface epithelium as well as in the stroma [58]. Differential mRNA expression of all three isoforms were found in fallopian tube epithelial cells with TGFβ1 mostly detected in epithelial cells and TGFβ2/3 equally expressed in both epithelial and non-epithelial cells [59]. TGFβ1 and 2 have demonstrated roles in ovarian cancers, however, it is not clear if TGFβ3 plays a direct role in EOC despite a few lines of evidence indicating potential roles in ovarian angiogenesis [60]. While other members including BMPs, Activins and Inhibins’ have important physiological and pathological roles in the ovary and in ovarian cancer [61–65], here, we focus solely on the TGFβ isoforms.

### Signal transduction mechanisms of TGFβ

Signaling and response to TGFβs’ is regulated at multiple levels, including ligand synthesis and activation [66, 67], presence of agonists and antagonists [68, 69] and cell surface receptors and co-receptors expression [44, 70]. The mature forms of TGFβ1-3 have 97% similarity and hence, exhibit redundancy in binding receptors [71, 72]. However, conformational differences exist between TGFβ3 and TGFβ1/2. TGFβ3 is structurally either open or exists as a mixture of closed and open conformations, which leads to more flexible ligand receptor interactions, while TGFβ1 and TGFβ2 are found largely in closed conformations. These differences are thought to play a role in differential biological functions of TGFβ3 as they may impact signal duration and amplitude, a key component in determining TGFβ signaling outcomes [73, 74].

#### The receptors and SMAD dependent transcriptional control

**Fig. 1:**
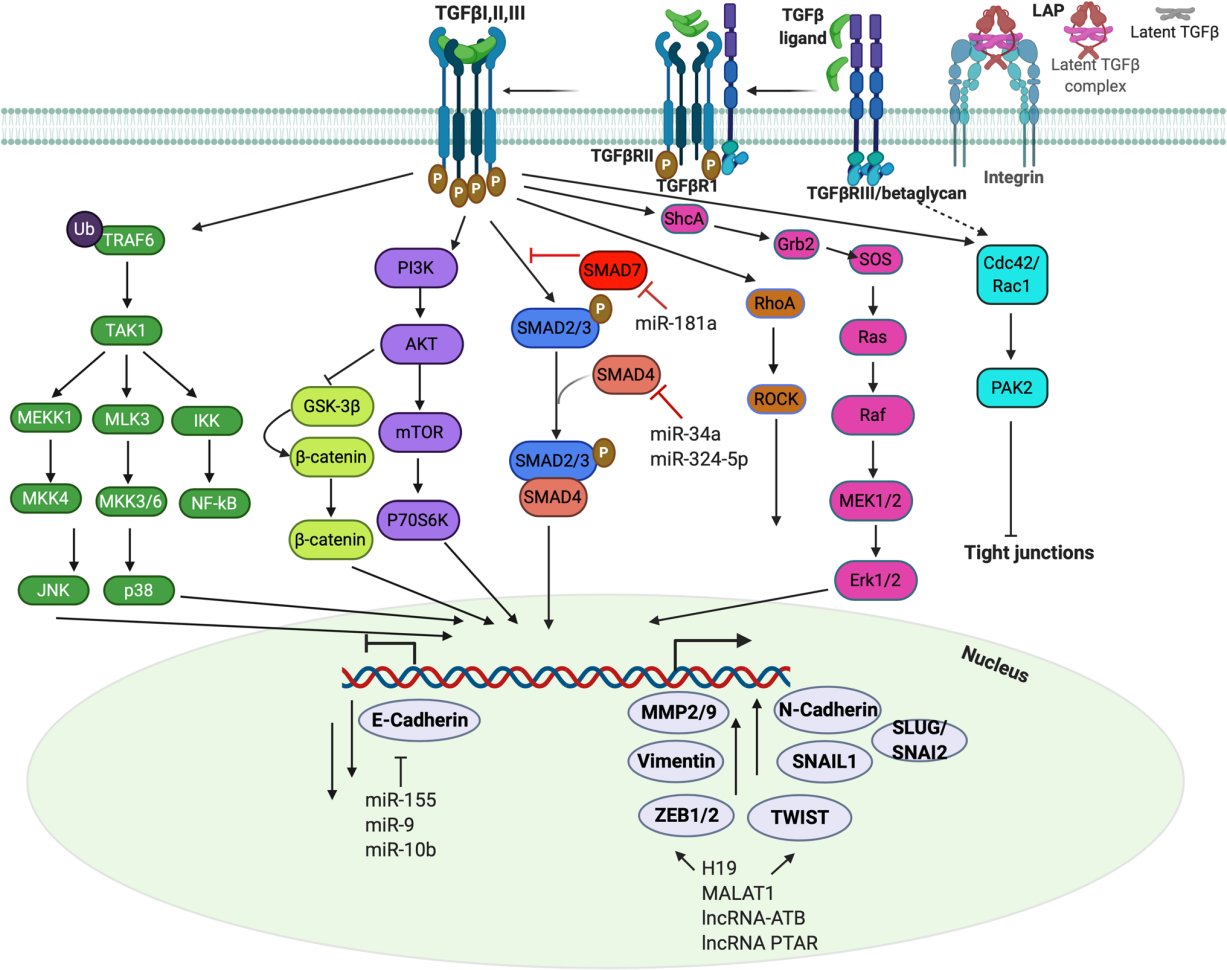
TGFβ signaling pathways in EMT. Cleavage of the pro-domain latency-associated peptide (LAP) releases active TGFβ that can bind cell surface receptors. Cell surface receptors include the Type III receptor (TGFβRIII/betaglycan), Type II receptor (TGFβRII) and the Type I receptor (TGFβRI) (also see Table 1). TGFβ elicits cellular responses by forming ligand-receptor ternary complexes. Constitutively active TGFβRII transphosphorylates TGFβRI on Ser-thr residues, activating its kinase activity, which in turn phosphorylates SMAD2/3 (blue). Phosphorylated SMAD2/3 forms heterocomplexes (heterotrimeric or dimeric) with SMAD4 and accumulates in the nucleus to regulate expression of genes associated with EMT. SMAD7 (red) terminates signaling by increasing turnover of the kinase receptors. TGFβ also mediates cellular responses via alternate signaling pathways including (from L-R) TAK1 activation by TGFβRI mediated TRAF6 ubiquitination that can induce NF-κB, JNK, p38MAP kinase signaling; induction of PI3K and AKT-mTOR signaling; TGFβ also regulates the WNT/β-catenin pathway via AKT inhibition of GSK-3β, releasing β-catenin for nuclear accumulation; TGFβ induces activation of RhoA-ROCK signaling; activates MEK/ERK pathway via phosphorylation of ShcA by TGFβRI leading to Ras activation and downstream MAP Kinases; TGFβ promotes interaction between CDC42/RAC1 and PAK2. Activation of TGFβ signaling either via SMAD or alternate pathways can induce expression of several EMT-TFs such as *TWIST, SNAIL, ZEB* to promote EMT and lead to repression of E-Cadherin. miRNAs and lncRNAs also play a role in TGFβ mediated EMT by either inhibiting or stimulating EMT. miR-34a, -324-5p antagonizes TGFβ-SMAD induction of EMT whereas miR-155, −9, −10b, −181a activate EMT

Cellular response to TGFβ occurs via an extensively characterized and defined cascade of events that are initiated upon binding of ligand to specific receptors [44, 75]. The TGFβ family kinase receptors are all single-pass membrane spanning serine-threonine kinase receptors, consisting of two subfamilies: Type I and Type II receptors (summarized in Table 1). Despite the large number of TGFβ superfamily members, the number of receptors is limited to five type II and seven type I (ALK1-7) receptors that are essential for signaling but can also exist in multiple heteromeric combinations [62, 75, 76]. An additional class of receptors are the co-receptors/Type III receptors (endoglin and betaglycan) that also bind ligand but act as either ligand reservoirs, modulate intracellular trafficking of internalized ligand and receptor, or increase affinity of the ligand for the serine threonine kinases [70] implicating them as critical for fine tuning signaling responses. Betaglycan and endoglin act as co-receptors for all three isoforms of TGFβ although betaglycan shows higher affinity to TGFβ2 and endoglin to TGFβ1 and 3 [77–79]. Within the heteromeric receptor complexes, the type II receptors phosphorylate and activate the type I receptors [80]. This activation initiates signaling via the SMAD-dependent pathways with the phosphorylated GS-domain on TGFβRI serving as a docking site for the receptor-regulated SMAD proteins (R-SMADs), allowing the specific recognition and phosphorylation of the R-SMADs at the SSXS motif in their carboxyl-termini (Fig. 1). The SMAD proteins can be classified as R-SMADs (SMAD1, 2, 3, 5, 8, 9); common-partner SMAD (SMAD4), and the inhibitory SMADs (SMAD6 and 7). The inhibitory SMADs antagonize intracellular signaling through interactions with the activated receptors and R-SMADs and increasing their degradation (Fig. 1). SMAD6 preferentially inhibits BMP signaling [81] while SMAD7 inhibits both the TGFβ and BMP signaling axes [82].

The type I receptor mediates signaling into either of two distinct R-SMAD pathways: TGF-β-SMAD pathway utilizes SMAD2/3 while the BMP-SMAD pathway utilizes SMAD1/5/8 [83]. However significant recent evidence [76, 84, 85] indicates that these SMADs are not exclusive to TGFβ or BMP respectively, adding to the complexity of responses. Phosphorylated complexes of SMAD2/3 or SMAD1/5/8 form a higher-order complex with the co-SMAD4 which then accumulates in the nucleus and binds to regions on the DNA to control transcription of several target genes (Fig. 1). The identification of the membrane receptors and SMAD proteins and analyses of the signaling kinetics in detail [86] have revealed that the diverse cellular responses generated by TGFβ in cells, do not necessarily connote the use of different signaling pathways, but rather, the different interpretation of outputs from the same signaling pathway.

Both the R-SMADs and the co-SMAD (SMAD4) have two conserved Mad homology domains (MH1 and MH2) at the amino and carboxyl terminus respectively [87] separated by a linker region. All R-SMADs except for SMAD2 can bind directly to DNA, via the MH1 domain, although their affinity for DNA is relatively low (*K*_*d*_ ≈ 1 × 10^−7^ M) [87] compared to other sequence specific transcription factors. SMADs bind to short sequences (SMAD-binding element SBEs) [87] and it is worth stating that a single SBE is not sufficient to recruit an activated SMAD complex. Due to this weak affinity of SMADs to the DNA, specificity of recruitment to DNA usually requires additional protein binding interacting partners via the MH2 domain including co-activators and co-repressors to drive the activation/repression transcriptional program [86, 88] thereby contributing to the contextual responses to TGFβs [89]. Both in the cytoplasm and in the nucleus, the SMAD proteins also undergo additional phosphorylation events in their linker regions that enables peak transcriptional activity including the phosphorylation by cyclin-dependent kinase 8/9 (CDK8/9) a component of the RNA POLII mediator complex and glycogen synthase kinase-3 (GSK3), resulting in the recruitment of YAP and PIN1 respectively to promote transcription and SMAD turnover [90–92]. Notably, CDK8 and YAP1 are required for EMT responses and TGFβ dependent metastasis in multiple models [93, 94]. Thus, TGFβ signals are interpreted in different ways resulting in the diverse responses depending on the cellular context, particularly relevant to the pathophysiology of cancer and EMT. Much has also been discussed about TGFβ and its co-operation with other signaling pathways including the Ras-MAPK and Wnt pathways in promoting EMT by increasing expression of the transcriptional repressors of E-cadherin TWIST, SNAIL and ZEB1 [20] (Fig. 1). Co-repressor complexes of SNAIL1 and SMAD4 can co-silence several tight junction proteins during EMT demonstrating SMAD dependency in EMT induction by TGFβ [95]. Much of the current research focus is on each individual TGFβ member, but the interplay between the different members in cancer and EMT remains to be fully elucidated.

#### SMAD dependent non-transcriptional mechanisms

Transcriptional control is the most investigated outcome of SMAD activation downstream of TGFβ, however significant SMAD functions in the cytoplasm have emerged particularly in RNA processing. SMADs have been implicated in RNA splicing, micro-RNA processing, as well as directly in miRNA mediated EMT (Fig. 1). For instance, miR-23a targets E-cadherin through the TGFβ/SMAD pathway to promote EMT [96]. Other miRNAs such as miR-155 [97], miR-9 [98] and miR10b [99] can also promote EMT via direct targeting of E-cadherin mRNA [100]. There are also numerous inhibitory miRNAs that counter the effect of TGFβ 1/2 on EMT; for example, miR-34a inhibits SMAD4 [101] and miR-324-5p suppresses TGFβ2 dependent EMT [102]. In ovarian cancer, the effect of a subset of miRNAs on EMT has also been well demonstrated as miR-181a promotes TGFβ mediated EMT via the repression of SMAD7 [103]. In contrast, miR-200s are highly expressed in ovarian cancer and correlate with an epithelial phenotype acting to inhibit EMT by targeting SMAD2 and SMAD3 [104]. A few additional mechanisms include roles for RNA binding proteins such as hnRNP E1(heterogeneous nuclear ribonucleoproteins) involved in mRNA processing events wherein, binding of a structural 33-nucleotide TGFβ-activated translation (BAT) element in the 3’Untranslated region of disabled-2 (Dab2) and interleukin-like EMT inducer (ILEI) transcript in response to TGFβ signaling promotes EMT [105].

Long non-coding RNAs (lncRNA) have also emerged as key regulators of TGFβ mediated EMT (Fig. 1) including H19 [106, 107], LINK-A [108] DNM30S [109] MALAT1 [110, 111], PVT1 [112] PE [113], and BORG [114–116]. Many act as sponges for miRNAs as in the case of H19, MALAT1, LncRNA-ATB and lncRNA PTAR that sponge miR-370-3p, miR-30a, miR-200 and miR-101-3p respectively to enhance either ZEB1/2 or TWIST1 expression during EMT or during EMT mediated metastatic outgrowth as in the case of BORG [110, 114–118]. MALAT1, which is frequently upregulated in EOC, also partakes in EMT by interacting with EZH2 and by recruiting chromatin modifiers [119–121] and induces formation of a lncRNA-protein complex containing Smads, SETD2 and PPM1A phosphatase leading to dephosphorylation of Smad2/3 and termination of TGFβ/Smad signaling [111]. Lastly, an under investigated area of regulation of EMT by TGFβ in the cytoplasm is SMAD’s potential role in mitochondrial function [122] that has emerged as an important player in regulating ovarian cancer metastasis [123]. Specifically, SMAD2 interacts with mitofusin2 (MFN2) and Rab and Ras Interactor 1 (RIN1) to promote mitochondrial fusion [122]. Whether these specific interactions result in mitochondrial and /or metabolic alterations and EMT however remain to be tested.

#### SMAD independent pathways

TGFβs can also activate a series of non-SMAD signaling pathways with similar and/or delayed kinetics to the SMAD pathways in a context dependent manner [124]. Most commonly, these pathways are activated directly by the Type II and Type I receptors or through the Type III co- receptors [124–127]. In the context of EMT, mitogen-activated protein kinase (MAPK) pathways Jun-N terminal kinase (JNK), extracellular-signal-regulated kinases 1/2 (ERK1/ERK2), p38 and PI3K kinases; AKT/PKB pathway, small GTP-binding proteins, RhoA, Rac1 and CDC42, and mTOR; protein tyrosine kinases such as PTK2, SRC and ABL, and the NF-κB pathway and Wnt/β-catenin signaling pathway have been examined in TGFβ dependent EMT and are reviewed elsewhere in detail and are summarized in Fig. 1 [128–130]. An example is the activation of ERK MAP kinase by TGFβ1 via the phosphorylation of the scaffold protein ShcA by the Type I receptor ALK5 [131] or phosphorylation of the Type II receptor directly by Src [132] leading to Ras and MAPK activation [133]. Consistently, inhibition of ERK MAP kinases inhibits TGFβ induced EMT [134]. Other mechanisms include ubiquitination mechanisms that also depend on Type I receptor interactions with tumor necrosis factor receptor-associated factor 6 (TRAF6) and the subsequent activation of the MAPKKK TAK1 upstream activator of JNK and p38 [135, 136] which is required for TGFβ-induced EMT, in non-ovarian cancers [128, 137]. TGFβ2 has also been shown to utilize both SMAD and non SMAD mechanisms in a subset of cancers to promote EMT and invasion via autophagic responses [138, 139]. Much like the Type I receptor, the Type II receptor TGFβRII can also phosphorylate other proteins besides the TGFβ receptors to impact EMT. At the level of cell- cell junctions, TGFβ regulates RhoA activity through Par6 interactions with TGFβRI leading to TGFβRII mediated phosphorylation of Par6 [140] and subsequent RhoA degradation at tight junctions [140] (Fig. 1). Thus, the TGFβ serine threonine kinases can have substrates beyond the SMADs and TGFβ receptors and vice versa the receptors can be phosphorylated by a variety of kinases, that may be relevant during EMT and other process. Given that the Type II receptors of the TGFβ family have been identified in genomic studies as driver protein kinases in about 5–15% of cancers [141], identification of all their substrates will likely shed light on additional mechanisms including EMT in cancer.

### TGFβ alterations and sources in epithelial ovarian cancer

Mutation hotspots exist in genes that encode a subset of TGFβ ligands and receptors (*TGFβR2*, *AVCR2A, BMPR2*), and SMADs (*SMAD2, SMAD4*) in many non-gynecological cancers [142]. In high grade serous EOCs’ (HGS), amplification frequency of the TGFβ pathway components listed above was found to be high, consistent with high genomic instability of these EOCs [142–144]. In the fallopian tube which is one of the sites of tumor initiation and early metastasis of HGS cancers, all three TGFβ isoforms and their receptors are expressed, with most reports indicating elevation of all three isoforms in primary, metastatic and recurrent EOCs compared to normal ovaries [58, 59]. However, a clear prognostic value for these changes has only recently emerged with increased access to genomic data, publicly available data sets and tools for investigators to analyze these including TCGA, KMplotter, Oncomine and DepMap to indicate a few. Such studies have revealed lack of a robust correlation between TGFβ1 expression and survival outcomes in women with ovarian cancer. However most notably, increased TGFβ2 and TGFβ3 mRNA expression were associated with poorer prognosis based on worse progression-free survival (PFS) and reduced overall survival (OS) [145]. While the utility of the TGFβ ligand expression as a biomarker continues to be debated, there is significant evidence that all isoforms are produced albeit at different locations and to different degrees. Indeed the source of TGFβ in ovarian cancers has been reported to be not just the tumor cells, but also the peritoneal mesothelium and tumor infiltrating cells [146]. Thus, understanding the specialized local sources and mechanisms of latent TGFβ activation during metastasis is likely more relevant to delineating specific TGFβ dependent outcomes in ovarian cancer.

## EMT and metastasis in epithelial ovarian cancer

### Ovarian cancer subtypes and metastatic route

**Fig. 2:**
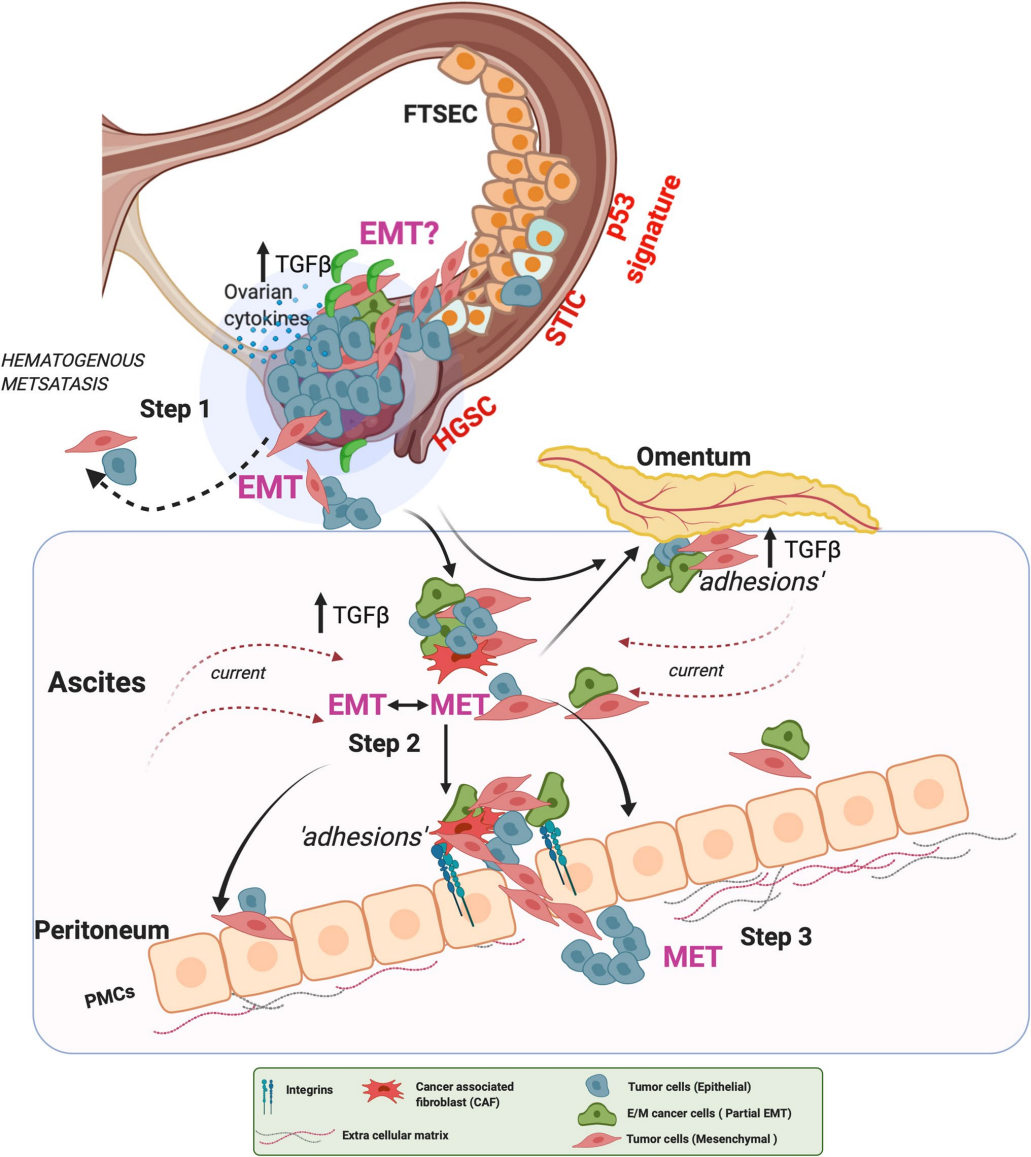
EMT events in ovarian cancer metastasis. In *step 1*. FTSECs in the fallopian tube develop STIC lesions with characteristic alterations in *TP53* that develop into HGS cancers in the fallopian tube and the ovaries. Epithelial ovarian cancer (EOC) cells detach and shed into the peritoneal fluid for transcoelomic spread or enter the blood vessels leading to hematogenous metastasis. In *Step 2*, shed EOCs in the ascites retain epithelial characteristics, undergo EMT, or acquire mesenchymal characteristics, or enter a partial E/M state, forming anoikis resistant cell aggregates. Ascites flow facilitates aggregate attachment and spread throughout the peritoneal cavity leading to cell aggregate ‘*adhesions*’ to the peritoneal membrane that covers the visceral organs and pelvic and abdominal cavities. Such adhesions in *Step 3* can undergo MET (reverse EMT) to acquire an epithelial phenotype enabling the cells to establish and grow at secondary sites including at the omentum. At the peritoneal interface, cancer cells invade PMCs facilitated by integrins and TGFβ, developing secondary tumors and metastasis. FTSEC - fallopian tube secretory epithelial cells, STIC - serous tubal intraepithelial carcinoma, HGSC - high grade serous cancer, EOC - epithelial ovarian cancer, MET - Mesenchymal to epithelial transition, PMCs - peritoneal mesothelial cells, TGFβ - Transforming growth factor-β

Ovarian cancer remains one of the leading causes of cancer related deaths in women accounting for 4.4% mortality worldwide [147] in part due to inadequate prevention and detection methods, and ineffective and insufficient therapies for advanced stage patients (Stage III or Stage IV). Ovarian cancers can be classified based on their cell of origin as either epithelial, germ or stromal type [148]. Epithelial tumors are more common in the population and include low and high grade serous (HGS), endometrial, clear cell and mucinous subtypes. Within the epithelial tumors, significant genomic heterogeneity exists and an in depth understanding of the differences between the subtypes is in fact required to improve precision medicine for these cancers [149]. The most common and aggressive subtype are the HGS, marked by a p53 mutational signature, early genetic instability and genomic heterogeneity [150, 151]. Gene expression profiling and subsequent clustering of these HGS cancers has led to the establishment of additional molecular subclasses that have been evaluated by TCGA as well [152]. In accordance with gene expression signatures, specific clusters were identified and divided into mesenchymal, immunoreactive, differentiated, and proliferative. In comparing survival outcomes, the immunoreactive subclass showed the best survival outcomes among all [153]. Additional classifications have also been proposed based on lesion size and spread in the peritoneum [154]. Notably, comparing EMT gene signatures revealed that peritoneal spread made up primarily of bigger implants correlated significantly with a reduced epithelial status as compared to widespread smaller lesions [154]. Of all patients diagnosed with serous ovarian carcinoma, ~ 15% have germline *BRCA *mutations [155]. Although *BRCA1* and *BRCA2* are intimately involved in DNA damage repair, direct links to TGFβ related EMT in ovarian cancer are emerging. A recent study in ovarian cancer reported that loss of endogenous BRCA1 dampens the tumor suppressive/growth inhibitory effect of TGFβ [156]. Several studies in breast cancer have established links through either BRCA1 dependent transcriptional regulation of EMT transcription factors, cytoskeletal proteins or micro RNAs which may indirectly support TGFβ dependent EMT [97]. Whether these mechanisms are active in ovarian cancers is currently unknown. Identifying the precise site of origin (ovary versus fallopian tube) and mapping the discrete metastatic steps in ovarian cancer has been challenging as compared to other cancer types. However, significant genetic and whole exome sequencing data point to the involvement of p53 mutated serous tubal intraepithelial carcinomas (STIC) lesions in the fallopian tube early on, with subsequent or continued metastasis into the fallopian tube epithelium, ovaries, peritoneum, omentum, uterus, pelvic walls and occasionally to the rectum and bladder [157–160] (Fig. 2). EOC metastasis is thus largely transcoelomic, with some evidence of hematogenous and lymphatic spread [161, 162] (Fig. 2). However, the contributions of hematogenous and lymphatic spread of EOC metastasis remain limited and somewhat controversial.

### Role of EMT in initiation of HGS EOC metastasis

EMT is part of the normal ovarian physiology during oocyte release [163]. Thus, it is likely that EOC tumor cells retain the capacity to transform and convert into a more mesenchymal state naturally and thereby invade into the peritoneum. One accepted model of peritoneal spread involves the detachment and shedding of cells from the tumor, which would likely require weakening of some cell–cell junctions and cell-ECM interactions (a hallmark of EMT) followed by survival under anchorage independence, re-attachment to new locations and establishment of new colonies within the transcoelomic/intraperitoneal cavity (Fig. 2). The detachment/shedding can be as single cells or clusters of cells as both are detected in the ascites of advanced EOC patients [164] (Fig. 2). Both cell survival and invasion events are associated with a mesenchymal gene and protein expression profile as cells that are able to grow under anchorage independence (a critical step during metastasis) exhibit a more mesenchymal phenotype, expressing high N-cadherin and *ZEB1,* and low E-cadherin [165, 166]. Whether EMT is a driver for initiating shedding is somewhat unclear. Immuno-histological analysis of both primary and metastatic ovarian carcinoma however reveal that EMT is significantly associated with peritoneal metastasis and correlated with low survival outcomes for ovarian cancer patients [167, 168]. Most recently, miR-181a, that can promote EMT by inhibiting SMAD7 (Fig. 1) has been reported to promote oncogenic transformation by increasing genomic instability in fallopian tube epithelial cells, in part through effects on the tumor suppressive *s*timulator of *i*nterferon *g*enes (STING) pathway [169]. STING and genome instability have in other cancers been reported to be associated with a mesenchymal signature and metastasis, wherein cells with high chromosome instability were enriched for EMT associated genes and pathways [170]. Thus, it is likely that the convergence of STING, chromosome instability and EMT factors, contribute both to cellular transformation and initiation of metastasis in ovarian cancers (Fig. 2). Here again, the precise source of TGFβ within the tumor in the fallopian tube or ovary, that may directly facilitate EMT needs to be defined as it may vary depending on the type of tumor, e.g., immunoreactive, versus mesenchymal or proliferative versus differentiated. Several lines of evidence indicate that within the tumor, local TGFβ secretion from infiltrating stromal cells, leukocytes, macrophages, and myeloid precursor cells could create a more favorable proinflammatory microenvironment for EMT [171]. For instance, release of active TGFβ via GARP produced from tolerogenic Treg cells has been shown to promote EMT and immune tolerance [172]. Similarly, TNF-α, which might be a result of infiltrating monocytes has been shown to promote EMT sensitivity as well [173]. A second environmental factor that could create an EMT conducive tumor is hypoxia, which either via HIF1/2, NFκB and/or changes in the redox environment has the potential to create and promote TGFβ dependent EMT leading to initiation of metastasis [174]. Similarly changes in the redox environment in the tumor has been shown to stimulate a p53/SMAD/p300 complex required for transcriptional increase in TGFβ itself which could be critical in HGS ovarian cancers that have a strong p53 mutational signature [175]. TGFβ has also been reported to be activated by mitochondria derived H_2_O_2_ both at the level of transcription and at the level of latent TGFβ activation in non-ovarian systems [176, 177]. While the roles for hypoxia inducible factors and changes in the redox environment in EMT and cooperation during metastasis is widely accepted, most of these studies as they relate to initiation of dissemination in ovarian cancer have been conducted in vitro and direct in vivo evidence at the stage of initiation of metastasis remains elusive.

## TGFβ and EMT in the metastatic epithelial ovarian cancer environment

### The peritoneum and the mesothelium

**Fig. 3:**
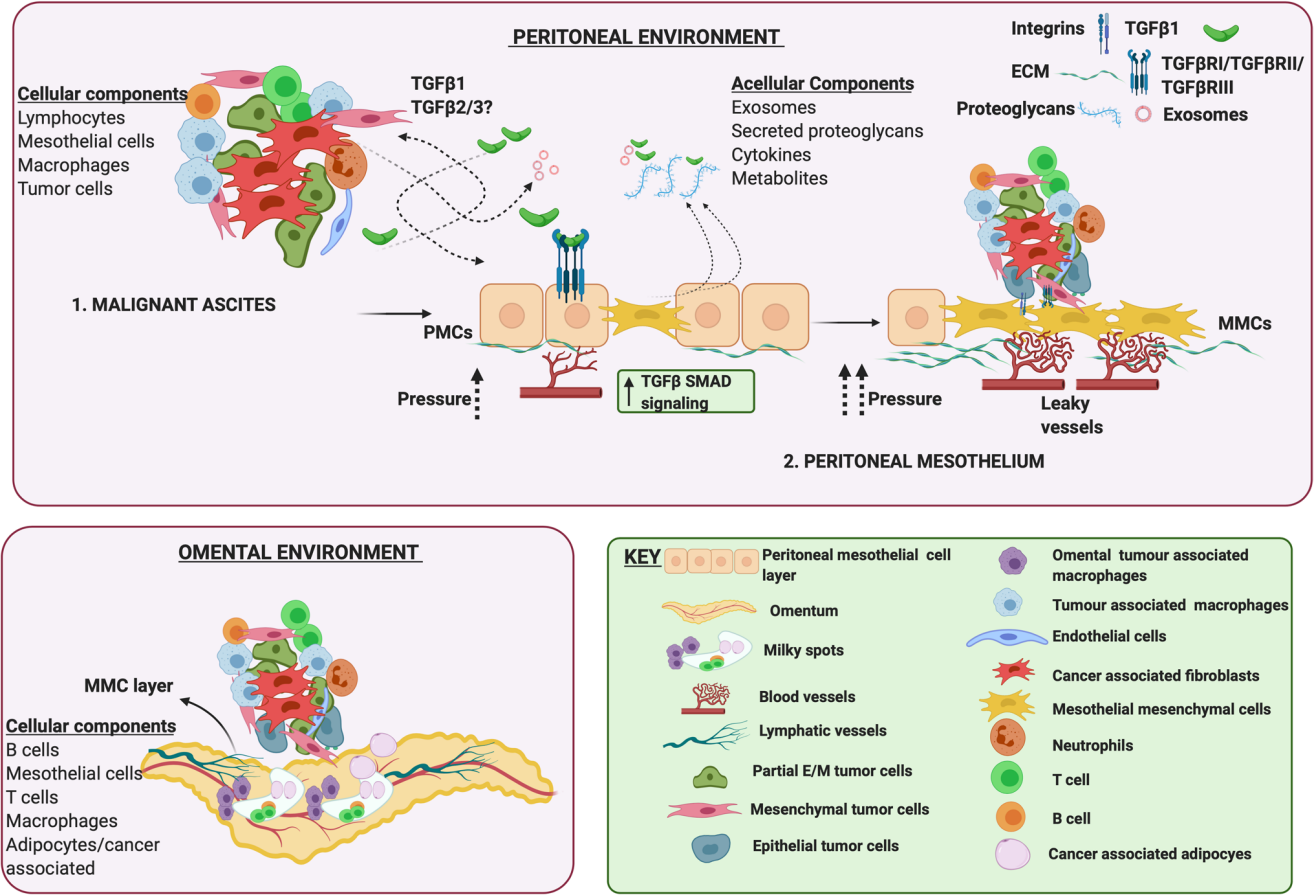
Ovarian cancer metastatic environment. The peritoneal and ascites environment are tightly linked to each other as leakage through the peritoneal mesothelium drives malignant ascites accumulation. *Malignant ascites* is composed tumor cells (in E, M or partial E/M states), either alone or in aggregates composed additionally of immune cells (macrophages, T cells, B cells and neutrophils), fibroblasts, and endothelial cells. Additional non cellular components include multiple cytokines such as TGFβ, exosomes that carry TGFβ, its receptors and also noncoding RNAs, metabolites and proteoglycans that are secreted primarily by the peritoneal mesothelial cells. In the *peritoneal mesothelium,* TGFβ1 released from tumor cells and CAFs can stimulate TGFβ/SMAD signaling in PMCs driving MMT, that can potentiate vascular changes leading to leakage and altered angiogenesis. Cell aggregates via integrins adhere to MMCs promoting metastasis by ECM degradation and vascular changes. The *omental environment* supports cell aggregate attachment to the omental MMCs, and growth preferentially near “milky” spots composed of lymphocytes, macrophages, and adipocytes. ECM - Extra cellular matrix, MMT - Mesothelial mesenchymal transition, PMCs - Peritoneal mesothelial cells, MMCs - Mesothelial mesenchymal cells, CAF - cancer associated fibroblasts

The peritoneum is a membrane of mesothelial cells, which lines the wall of the abdominal cavity, lying on abdominal and pelvic organs, including the omentum. The peritoneal mesothelial cells (PMCs) create a mechanical barrier for the abdominal organs. The peritoneal cavity acts as a rich *“soil”* of ECM proteins such as collagen I and other adhesion molecules that can support cell proliferation, migration and invasion. The peritoneum thus provides a site for EOC cells, aggregates, and clusters to attach and invade [178–180]. In this environment tumor cells have been shown to interact with the PMCs via TGFβ signaling, wherein cancer cell derived TGFβ1, via TGFβ1 receptor interactions on the PMCs’ activates a RAC1/SMAD pathway, leading to increased fibronectin expression and a mesenchymal phenotype in the PMCs [181]. Such studies establish the mesothelium as an active player in metastasis, with TGFβ and the mechanisms of EMT central to the invasion process. In this scenario, it is possible that cells, prior to attachment exist in mesenchymal or partial mesenchymal states (Fig. 2). These states could be acquired as a result of EMT during detachment from the primary tumor sites (Fig. 2) or acquired while under anchorage independence as a result of inter-cellular interactions (Fig. 3), thereby priming the cells for a complete EMT transition and effective peritoneal invasion. Kinetic models of EMT that incorporate multiple states of EMT [182] will be required to address this in detail in the future.

A variation on the classic cancer EMT of tumor cells is mesothelial-mesenchymal transition (MMT) of the PMCs, wherein PMCs transition into mesenchymal cells (MCs), acquiring migratory and fibroblast like phenotypes [183], much like cancer associated fibroblast (CAFs). MMT has been extensively investigated in other tissues during fibrosis of the peritoneal membrane and in cancers such as mesothelioma [184]. The “activation”/MMT process could thus play an active role in facilitating metastasis as TGFβ is also produced by the peritoneum itself [185]. Partnering with EOC cells to invade the mesothelium are CAFs. CAFs have been shown to mediate invasion through the sub-mesothelial layer by producing ECM components and growth factors turning the peritoneal cavity into a metastasis conducive niche for EOC cell attachment and metastasis [186]. Additional mechanisms of peritoneal invasion utilize integrins. Of note CAFs in the ascites of EOC patients are present in aggregates with tumor cell clusters that express integrin α5 and are highly efficient as a unit at invading and metastasizing into the peritoneum as a result of TGFβ signaling activation [187]. Increased expression of β1-integrin has also been shown to result in direct cell–cell interactions between EOCs and PMCs as confirmed by electron microscopy and adhesion studies [186]. Thus, while several studies have demonstrated the role of mechanical ‘pushing’ forces where cell clusters in the form of spheroids can impose integrin and myosin generated forces to invade the mesothelial layer, destruction of the mesothelium may be a prerequisite for invasion. Indeed, tumor induced apoptosis of the mesothelium via the Fas/FasL pathway has been shown to promote PMC clearance and invasion at the initial stages of metastasis [188, 189]. Whether TGFβ plays a direct role in dictating both EMT and apoptosis in the mesothelium is worth speculating, as TGFβ can in fact concurrently induce both apoptosis and EMT as demonstrated in pancreatic and liver cancers [190, 191] and perhaps in other systems as well. Lastly, but far from the least, the role of the immune environment in peritoneal spread of EOCs is critical as depending on the number and size of peritoneal lesions, the peritoneal microenvironment can present either a more adaptive response or a more systemic inflammatory response [192].

### The ascites

As EOC progresses, metastatic fluid (ascites) accumulates in the peritoneum. Increasing volumes of ascites are thought to generate a favorable tumor microenvironment, enabling transcoelomic tumor spread in a feed forward manner. As such ascitic volume and components have been used to grade, predict stage and survival outcomes including chemoresistance outcomes of EOC patients. Some studies also suggest ascites to be an independent prognostic factor in EOC [193–195]. Ascites can also contribute to morbidity due to gastrointestinal problems and while in most cases treating the underlying disease will reduce ascites, untreatable ascites can be a recurrent and frequent problem requiring drainage and paracentesis. Ascites accumulation in EOCs has been attributed to multiple factors including increased vascular permeability of vessels lining the peritoneum, lymphatic obstruction leading to reduced lymph drainage and also angiogenesis triggered by tumor cells and CAFs attached to the peritoneal wall [196, 197]. In this context, TGFβ blockade prevents destruction of the lymphatic vessels leading to control of ascites acting in part through VEGF inhibition [198]. TGFβ1 that has been reported to be elevated in the ascites of EOC patients [199, 200]. In-depth analysis of the other TGFβ isoforms is currently lacking and this may further shed light on TGFβ2/3’s role as well in the future.

The ascites constitutes its own environmental niche (Fig. 3) as it not only constitutes much of the same cellular components from the primary tumors such as the tumor cells, immune cells, fibroblasts, endothelial cells and lymphocytes but also harbors mesothelial cells [201] and likely tumor cells shed from metastatic sites as a result of forces from the ascites current (Figs. 2, 3). In general, approximately 37% of the cellular composition of the ascites constitutes lymphocytes, about 30% macrophages and mesothelial cells and 0.1–0.5% constitutes carcinoma cells [201] and the remaining includes fibroblasts and endothelial cells. A recent single cell analysis that examined EMT signatures of single and aggregate tumor cells from the ascites not only confirmed the heterogeneous mix of the ascites, but also demonstrated a strong EMT program that was dependent on the CAFs and notably TGFβ [202]. These findings are also consistent with prior reports demonstrating the impact of the ascites on inducing EMT in cell line models [203]. Nevertheless, not just would EMT of the cells in the ascites promote peritoneal invasion and metastasis, but also contribute to anoikis resistance. While much has been debated on whether cells under anchorage independence in the ascites are truly matrix detached, survival in the ascites requires all the cells in the environment to have adapted to changes in matrix attachment. Nonetheless, anoikis resistance is an accepted pre- requisite of malignancy [204] and has been strongly linked to an EMT signature [166].

In addition to the heterogeneous cellular population in the ascites that can produce and respond to TGFβ, several recent studies have also characterized the presence of exosomes (Fig. 3) that have been proposed as biomarkers in EOC as they appear to correlate with tumor progression [205]. Notably TGFβ1 and receptors for TGFβ are known cargos of exosomes [206] and it is thus highly likely, that either TGFβ itself or components of the TGFβ signaling machinery may be delivered to tumor cells to impact EMT. Exosomes have also been shown to directly impact EMT in tumor cells [207] via miRNAs and lncRNAs [208] and hence can serve as a way to increase intracellular communication within the ascites [209] and thereby promote invasiveness. Another cargo of the exosomes includes proteoglycans [210] that are known regulators of TGFβ (e.g., decorin and biglycan) and are synthesized and secreted from the peritoneum [211], perhaps into the ascites where they can control TGFβ signaling (Fig. 3). Whether cells in the ascites undergo EMT at the primary tumor prior to shedding (Fig. 2) or acquire EMT characteristics in the ascites remains to be determined and exosomes could play pivotal roles in regulating this process.

### The omentum

The omentum is a specialized adipose tissue in the peritoneal cavity and is also the most preferred metastatic site for HGS cancers [212]. Acquisition of EMT characteristics has been shown to depend on cells in the omentum in mouse models [213]. However, in clinical practice, performance of omentectomies in patients without bulky disease or a grossly normal omentum has not yet been definitively shown to improve survival [214]. The omental microenvironment is undoubtedly highly conducive for tumor growth via both metabolic and immunological factors as evidenced by advanced EOC patients exhibiting significant omental tumor load. The omentum has also emerged as a pre-metastatic niche for progression and development of invasive HGS cancers [215]. The omentum is a highly vascularized tissue and contains ‘milky spots’ (in both humans and mice) which are primarily aggregates of leukocytes referred to as fat-associated lymphoid clusters (FALCs) [216] (Fig. 3). The lymphatics of the omentum serve as a conduit for fluid drainage from the peritoneum making it an ideal spot for tumor cells to land. Recent studies have used 3D coculture models indicating that EOC tumor cells via TGFβ can stimulate activation and proliferation of omental resident fibroblasts that in turn stimulates cancer cell adhesion, invasion and peritoneal dissemination [217]. In a corollary fashion, a ten gene signature that includes collagen-remodeling genes regulated by TGFβ1 signaling has been correlated with increased metastasis and poor patient survival [218]. This is particularly significant as the omentum is collagen rich and serves as a robust site for tumor cell adhesion via integrins [219].

Analogous to the peritoneum, mesothelial cells in the omentum can also secrete TGFβ that impacts the fibroblasts and tumor cells, and also the immune state of the omentum [220]. The omentum also hosts a unique macrophage population, expressing CD163 and Tim4 that can interact with EOC cells to promote metastasis [213]. Tumor Associated macrophages (TAMs) are a significant source of TGFβ and other EMT mediators [221–223] playing key roles in creating an immune suppressive environment in the omentum [215, 220]. Thus several recent studies have focused on understanding the immune microenvironment of the milky spots in the omentum [220]. Adipocytes are the other cell type highly enriched in the omentum that have been shown to have a symbiotic relationship with EOCs, and are coined cancer associated adipocytes (CAA) [224] (Fig. 3).These CAAs can act as powerhouses during advanced disease, providing the necessary energy for rapidly growing tumor cells via FABP4 [212], a chaperone for free fatty acids. FABP4 levels are indicators of increased residual disease after primary debulking surgery of advanced HGS patients [225]. In some in vitro systems FABP4 has been shown to promote EMT via TGFβ [226].Other CAA derived chemokines have been shown to support tumor progression by inducing a partial EMT in breast cancer models [227] and can cooperate with endotrophin, a cleavage product of collagen VI α3 chain to promote EMT [228]. Worth noting is the role of TGFβ as a strong negative regulator of adipogenesis, acting via non-SMAD mechanisms in breast cancer [229]. In the same study [229] adipogenesis induction reduced invasiveness with the CAAs localized to tumor borders [229]. The interplay between adipogenesis and EMT in the omentum of metastasized HGS ovarian cancer patients remains to be determined.

## Targeting TGFβ and EMT for EOC management

### EOC specific challenges

Complete debulking surgery is commonly the first- line of treatment for EOCs, followed up with a carboplatin and paclitaxel chemotherapy regimen. Most EOC patient tumors fall under one of two categories, either they are chemo-resistant at the outset, or will eventually become chemo-resistant. Thus, management of recurrent and resistant disease is one of the biggest challenges for EOC. Approximately 50% of EOC patients who have alterations in BRCA1/2, and/or alterations in other homologous recombination repair deficient pathways (HRD genes), are more likely to be carboplatin sensitive. For such patients, PARP inhibitors hold great promise with significant increases in PFS reported [230]. However, there is a dearth of treatment options for the remaining 50% of patients. Most of these patients respond well initially, but more than 70–80% of patients overall will relapse in less than 5 years [231] regardless of the original response status. Thus, disease management to improve and prolong survival remains a continuous challenge.

**Fig. 4:**
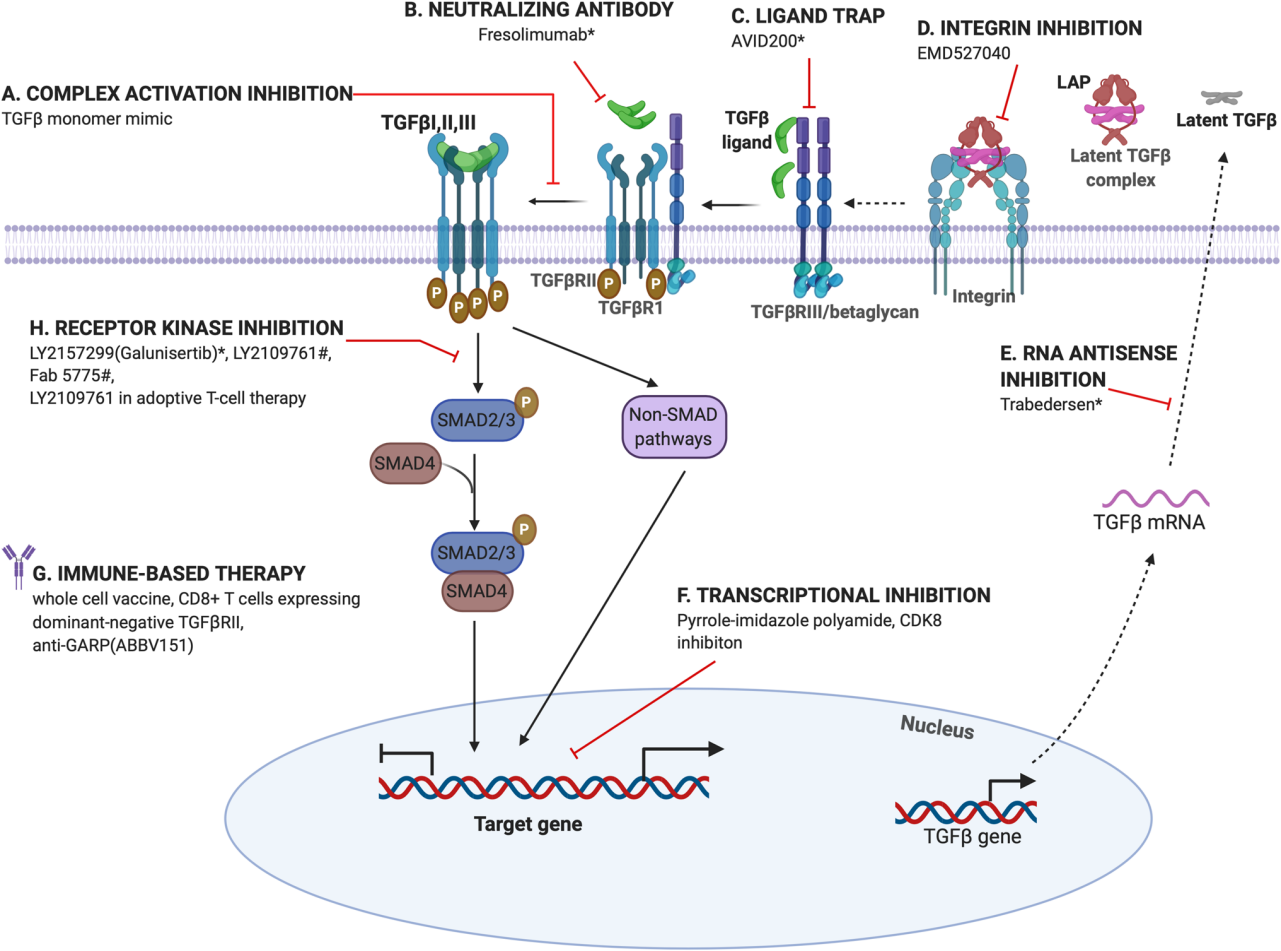
Therapeutic strategies targeting TGFβ signaling. Approaches both at the preclinical and clinical stage (see Table 2) are included to demonstrate points of inhibition. TGFβ signaling can be targeted using antibodies blocking TGFβ receptor-ligand interactions, TGFβ ligand neutralizing antibodies, soluble receptor ectodomain constructs to sequester ligands (ligand trap), small molecule inhibitors against TGFβRI receptor kinase activity, anti-integrin and anti-GARP, inhibition of TGFβ activation, RNA antisense oligonucleotides preventing TGFβ translation and at the transcriptional level using peptide inhibitors and CDK8 inhibitors

Chemo-resistant tumors express a more mesenchymal gene signature that also coincides with stem cell like features [232]. Notably, at the completion of primary platinum-based chemotherapy, HGS ovarian cancer patients were found to express high levels of cancer stem cell (CSCs) markers such as CD44 (a non-kinase transmembrane glycoprotein), CD133 or prominin-1, Aldehyde Dehydrogenase 1 Family Member A1 (ALDH1A1) as compared to primary tumors. Thus CSC enrichment during courses of chemotherapy may have contributed to resistance and relapse [233]. Several molecular mechanisms have been proposed for resistance acquisition by CSCs such as autophagy for survival by recycling metabolites [234], high expression of ATP-binding cassette (ABC) transporters to increase drug efflux [235, 236] and increased DNA polymerase η (Pol η) synthesis to compensate for drug induced DNA damage [237]. Several of these mechanisms are in fact downstream of EMT [238] and EMT transcription factors. Specifically, the EMT transcription factor SNAIL has been shown to be required to maintain stem like features in multiple ovarian cancer models [239, 240] in part via tumor suppressor miRNA let-7 [239]. ZEB1 has also been shown to promote EMT and stemness by increasing SOX2, OCT4, NANOG, CD44, and CD117 expression resulting in resistance to cisplatin [241]. Such studies strongly suggest TGFβ induced EMT as a mechanism to promote stemness in ovarian cancers. In breast cancers, increased TGFβ signaling via increased cell surface receptor expression has been directly linked to chemoresistance [242]. These cells can be resensitized to chemotherapy using Galunisertib or LY21567299 (small molecule TGFβR1 inhibitor (Fig. 4 and [242]). Whether this approach is effective in EOCs remains to be determined.

### Targeting EMT

Being able to target EMT related pathways has been a landmark development for cancer therapies. Several approaches and therapeutic targets have been developed or are being evaluated [243] including in EOC (Table 2). EMT itself can be targeted at multiple levels including, (a) blocking signals that induce EMT (including TGFβ inhibitors (see section below)), (b) blocking downstream transducers (such as tyrosine kinases and miRNAs) [244] (c) blocking mesenchymal mediators such as fibronectin, vimentin and N- cadherin (such as Artemisinin that reduces vimentin expression and can reverse EMT) [245, 246] and (d) blocking the MET transition which has been shown to be mediated by other members of the TGFβ family particularly BMP7 [247, 248]. Additional approaches include exploiting tumor vulnerabilities that arise as a result of EMT. One example is the exploitation of the plasticity and programmability of cells undergoing EMT as seen in breast cancer models wherein cells were forced to transdifferentiate into adipocytes, which in turn reduced metastasis [249]. An additional dependency that has garnered significant attention is metabolic adaptation to meet increased energy demands during cancer metastasis [250, 251]. One candidate with promise in EOC, is metformin, which in preclinical models can inhibit EMT, stemness and reverse chemotherapy resistance. Most notably, in a recent Phase II study with HGS patients, metformin was found to be well tolerated and reduced the stem cell population in these patient tumors. Improved OS was also observed, prompting Phase III studies for the future [252]. Thus, targeting ovarian cancer with standard of care in combination with metabolic inhibitors could suppress EMT related progression and yield promising results.

**Table 2:** Summary of TGFβ targeting drugs in clinical trials

Name	Mechanism of action	Cancer type	Clinical trial identifier number	Ovarian cancer patient recruitment	Reference
Galunisertib (LY2157299)	TGFβRI kinase inhibitor	Advanced Hepatocellular carcinoma	NCT02240433	No	[282]
Fresolimumab (GC10080)	Anti-TGFβ monoclonal antibody	Advanced malignant melanoma or renal cell carcinoma	NCT00356460	No	[283]
TβM1	Anti-TGFβ1 monoclonal antibody	Adenocarcinoma of the colon	–	No	[284]
NIS793	Anti TGFβ antibody	Advanced malignancies	NCT02947165	No	[285]
Trabedersen (AP12009 or OT-101)	Synthetic TGFβ2 antisense oligodeoxynucleotide	Glioblastoma multiforme or Anaplastic astrocytoma	NCT00431561	No	[286]
ABBV151	GARP binding, which interferes with production and release of active TGF β Tregs	Advanced Solid tumors	NCT03821935	Yes	[287]
STM 434	Soluble receptor ligand trap targeting Activin A	Ovarian Cancer( granulosa cell tumors) and other Advanced solid tumors	NCT02262455	No	[265]
AVID200	TGFβ1 and TGFβ 3 neutralizing antibody	Malignant Solid Tumors	NCT03834662	No	[288]
Vactosertib (TEW-7197)	TGFβR1/ALK5 inhibitor	Advanced solid tumors	NCT02160106	Yes	[289]
PF-03446962	ALK1 inhibitor	Advanced solid tumors	NCT00557856	Yes	[290]
Bintrafuspalfa (M7824)	Bifunctional fusion protein that sequesters TGFβ and blocks PD-L1	Non-small cell lung cancer, HER2 positive breast cancer	NCT02517398	No	[278]
Belagenpumatucel-L (Lucanix)	TGFβ2 antisense modified non-viral based allogenic tumor cell vaccine	Non-small-cell lung cancer at different stages	NCT00676507	No	[291]
TAG vaccine	Vector co-expressing GM-CSF	Advanced metastatic	NCT00684294	Yes	[292]

### TGFβ *specific targeting strategies and clinical progress*

TGFβ specific targeting can occur at multiple steps (Fig. 4) including (a) at the level of TGFβ ligand activation and directly limiting ligand availability (b) at the level of receptor inhibition and blocking receptor kinase activity (c) inhibition of transcription regulation by SMADs (d) indirectly by immune based strategies. Several antibodies, peptides, small molecule inhibitors and receptor trap-based approaches have been developed many of which are in clinical trials (Fig. 4 and Table 2). However, given the discovery of TGFβs in the late 1970s, progress to the clinic has been relatively slow, in part due to the discovery of cardiac toxicity in dogs after continuous administration of a small molecule inhibitor to TGFβR1 [253] and the complex roles of TGFβ itself in cancer progression acting either as a tumor suppressor or tumor promoter in a context dependent manner. Defining the source and role of TGFβ particularly in the stroma to promote EMT and metastasis, and in the distinct immune cell types, including CD4+ , CD8+T cells, NK or dendritic cells [254], has provided much renewed faith in therapeutic targeting of TGFβ in cancer.

#### Ligand control

TGFβ ligand neutralizing antibodies and antibodies that block TGFβ receptor ligand interactions have received significant pharmaceutical and clinical attention, however with limited progress specifically in ovarian cancers. Fresolimumab ([255] (Sanofi, GC1008) is a high affinity fully human monoclonal antibody that is a pan neutralizing antibody for all three TGFβ isoforms with promising clinical findings in non-ovarian cancers and particularly breast cancer patients receiving radiotherapy [255, 256]. Fresolimumab is also being explored for the treatment of Osteogenesis Imperfecta (OI) as animal model studies indicate that silencing TGFβ increases bone mass [257]. The findings from the OI trials could have beneficial side effects for the management of ovarian cancer patients as well who are mostly menopausal and over time suffer significant bone mass loss [258].Trabedersen (AP12009 or OT-101) has also emerged as an alternative strategy, which is an antisense approach targeting TGFβ specifically and has shown significant promise in pre-clinical ovarian cancer models [259]. Another approach to block TGFβ at the ligand level is to block activation using anti-integrin approaches [260] and anti-GARP (ABBV151) approaches [261]. GARP is specifically required for TGFβ activation in regulatory T cells (Tregs) and platelets and ABBV151 is currently in clinical trials (Table 2) in combination with immune checkpoint inhibitors (ICI). Other approaches in the research and development phases that have or are being explored include the development of a high affinity engineered TGFβ monomer that acts as a dominant negative due to its inability to dimerize with TGFβR1 and activate signaling, and a peptide ligand trap based approach that utilizes a sequence of the Type III TGFβ receptor (betaglycan) [262, 263]. The use of betaglycan based approaches is quite attractive as the domains by which it binds the different TGFβ members has been well mapped [264] providing potential for selective inhibition of not just the TGFβ isoforms but also beyond for other TGFβ members including Inhibins, Activins and BMPs [265]. Current TGFβ isoform specific traps include AVID200 that blocks TGFβ1 and TGFβ3 and is currently in clinical trials (Table 2). Thus, delineating isoform specific effects remains important for the long-term success of such agents in EOC and other cancers.

#### Receptor activation and transcriptional inhibitors

Small molecule-based approaches (Table 2, Fig. 4) have historically been the favorite approach for targeting TGFβ signaling at the reception level due to their ease of administration despite limitations of non-specificity of the compounds in some cases. Several of these compounds target either both the Type I and II receptors including LY2109761 or just the Type I receptor such as LY2157299 (galunisertib) and showed significant promise in pre-clinical studies in ovarian and other cancers where they were found to suppress metastasis and/or reduce cisplatin resistance [266, 267]. However, acquired resistance to LY2109761 was observed via increased TGFβ signaling after long term exposure [268]. Several additional compounds are currently in development [269]. Despite the progress and promise of galunisertib, it was discontinued early in 2020 (Jan 2020, Eli Lily news) for undisclosed reasons. Approaches targeting the Type II receptor specifically have been limited. One synthetic F’ab based inhibitor that emerged from a phage display screen to TGFβRII, has been evaluated in EOC models and was found to suppress metastasis through inhibition of EMT suggesting that the Type II receptor is also a feasible target for EOCs [270].Targeting at the transcriptional level remains rather underdeveloped, likely because of the lower affinity of SMADs’ for DNA and several non- SMAD mechanisms that are active. Indirect mechanisms include a pyrrole-imidazole polyamide drug at the level of TGFβ target gene transcription and other RNA (antisense) based blocking approaches to the mRNA for the different isoforms [271–275]. Similarly, transcriptional inhibition specifically of the metastatic and EMT based responses can be secured by inhibition of CDK8 in preclinical studies [93] [94]. CDK8 inhibitors are rapidly approaching the clinic and could hold great promise for ovarian cancers as well.

#### Immune options

Mechanisms to re-activate the immune system and improve the efficacy of immune checkpoint inhibitors (ICI) in cancers particularly ovarian cancers, is currently being actively explored as evidence in multiple other cancer studies indicates this may be an effective approach [276].

That the anti- metastatic effects of the TGFβ inhibitors are potentiated by this combination is also strongly based on prior studies that non-responders to checkpoint inhibitors have elevated levels of the central TGFβ pathway components (TGFβ1, Type I and II receptors) [277]. Supporting this, M7824 a bifunctional fusion protein composed of a monoclonal antibody against PD-L1 fused to the extracellular domain of TGFβRII, is preclinically effective at suppressing metastasis and also provided antitumor immunity [278]. M7824 is currently in clinical trials (Table 2) for multiple solid tumors. Preclinical studies using other approaches including the use of LY2109761 in adoptive T-cell therapy has also been proposed as a way to effectively increase immunotherapy efficacy [279]. Other approaches include whole cell vaccine based methods that use autologous tumor cells expressing an anti-sense to TGFβ2 [280] or tumor-specific CD8 + T cells modified to express a dominant-negative TGFβRII (non- ovarian cancers) [279]. Phase I trials of the vaccine approach have shown low toxicity and durable responses so far [281].

#### Future outlook with TGFβ based therapeutics in ovarian cancers.

Progress through the clinical pipelines for ovarian cancers has been slow with a major gap being lack of biomarkers to stratify patients and identify those who will benefit the most. This is a central issue for the progression of targeted therapies in EOC. With the acceptance that TGFβ inhibitors are likely to be most beneficial in combination therapy as opposed to as a monotherapy, either with immune checkpoint inhibitors or other approaches including anti- angiogenic strategies (not reviewed here), DNA intercalating agents and even with PARP inhibition, the outlook for the expanded use of TGFβ inhibitors in ovarian cancers remains positive.

## Concluding remarks

TGFβ signaling mechanisms have been examined for several decades. Yet fundamental insights both into the mechanism of action and the context of action continue to emerge. The recent advances outlined here have revealed the broad impact of TGFβ signaling mechanisms in EMT and cancer. These combined with our growing understanding of unique disease environments (such as discussed here for ovarian cancer) have provided new contextual information and understanding the detailed mechanisms by which plasticity is dictated will be critical for the future. With the advent of several TGFβ approaches in the clinic, such momentum is prescient. Emphasis should also be laid on understanding the interplay between the multiple TGFβ members that have in recent years emerged as playing key roles in cancer progression.

## Abbreviations

*TGFβ*: Transforming growth factor beta
